# Stabilization of *Ficus carica* L. Drink by utilizing varying levels of ultrasound-assisted moringa extract as a natural preservative

**DOI:** 10.1016/j.ultsonch.2024.107133

**Published:** 2024-10-28

**Authors:** Faiza Javed, Saima Tehseen, Faiza Ashfaq, Aysha Sameen, Waseem Khalid, Rizwana Batool, Ahmed Bilal, Muhammad Zubair Khalid, Tawfiq Alsulami, Robert Mugabi, Gulzar Ahmad Nayik

**Affiliations:** a Department of Food Science and Technology, Faculty of Science and Technology, Government College Women University Faisalabad, Faisalabad, Pakistan; b Department of Organic Chemistry, Faculty of Chemical Sciences and Technologies, University of Castilla La Mancha, 13071 Ciudad Real, Spain; c University Institute of Diet and Nutritional Sciences, Faculty of Allied Health Sciences, The University of Lahore, Lahore, Pakistan; d School of Agriculture, Food and Ecosystem Sciences (SAFES), Faculty of Science, Food Science and Nutrition (Food Chemistry), The University of Melbourne, Level 3, Building 194, Royal Parade, Parkville, Victoria 3010, Australia; e Department of Food Science, Government College University Faisalabad, 38000 Faisalabad, Pakistan; f Department of Food Science & Nutrition, College of Food and Agricultural Sciences, King Saud University, Riyadh 11451, Saudi Arabia; g Department of Food Technology and Nutrition, Makerere University, Kampala, Uganda; h Marwadi University Research Centre, Department of Microbiology, Marwadi University, Rajkot, Gujarat 360003, India; i Department of Molecular Food Chemistry and Food Development, Institute of Food and One Health, Gottfried Wilhelm Leibniz University Hannover, Hannover, Germany

**Keywords:** Fig, Moringa, Ultrasound extraction, Functional drink, Natural preservative

## Abstract

Fig fruit (*Ficus carica* L.) drink is a source of healthy minerals, vitamins, and bioactive ingredients however to improve the shelf-life of functional drink naturally, moringa leaf extract was compared with optimized concentration of potassium metabisulphite (synthetic preservative). Purposely, fig fruit drink, without preservatives was considered as negative control whereas, 0.2 % potassium metabisulphite-based fig fruit drink was taken as positive control. Further, ultrasound assisted extracts of moringa at varied levels; 5, 10, 15, and 20 % were incorporated in the fig fruit drink as natural preservative to test antioxidant, storage, and sensory quality against control samples. Resultantly, the maximum loss in antioxidant activity (18–38 %) and functional ingredients (24–56 %) was observed in negative control sample, in response to high microbial expansion till the termination of the study. Additionally, acceptability score for negative control sample was maximum at Day 1, that afterwards faced significant decline at 30th Day (6.6 ± 0.3). In contrast, positive control sample demonstrated minimum loss of free radical scavenging ability (7–22 %), polyphenols (11 %) and flavonoids (7 %) thus indicated maximum control on microbes i.e. 61–63 % as compared to negative control. Further, positive control sample indicated optimum consumer preference (7.0 ± 0.3) that remained stable throughout storage. Further, as the concentration of moringa exceeded from 5 to 20 %, the loss of functional ingredients reduced from 13 to 24 to 6–11 % and deterioration in antioxidant capacity suppressed from 14 to 26 to 8–20 %, correspondingly however, the sensory acceptability showed a declining trend, and 20 % moringa based sample portrayed poor consumer response (5.0 ± 0.2). Lastly, it was deduced that control on microbes was directly proportional to the concentration of moringa extract in fig fruit drink, that was poor in 5 % moringa extract concentration; 32–54 %. Conclusively, customer preference was reasonable (6 ± 0) at 15 % moringa extract concentration so this level should be employed in fig fruit drink for realistic control on bacterial (57 %) and fungal (47 %) activities.

## 1 Introduction

Fruit beverage industry in Pakistan is now booming rapidly and new versions of juices are brought into the focus of health-conscious consumers based on their positive health outcomes [1]. The nutritional benefits of fruit drinks are associated with its polyphenols and vitamin C, that help in detoxification mechanism as well as stabilize free radical chain reactions within the physiological system of the body [2], [3], [4].

In this regard, fig is a nutritious and tasty fruit, that belongs to the genus *Ficus carica* and family Moraceae. It is a rich source of vitamin A and C and minerals like Ca, Fe and K, along with amino acids, and organic acids. Further, fig fruit pulp contains cyanidin-3-O-rutinoside as the main anthocyanin, gallic acid & chlorogenic acid as phenolic acids and flavonoids encompassing rutin, quercetin-3-O-rutinoside & epicatechin [5], [6], [7], [8], [9], [10]. Based on these components, fig could prevent constipation and chronic anaemia and may resolve digestive, respiratory, and cardiovascular malfunctioning and owes anti-inflammatory traits as well [11]. Hence, introduction of fig drink into the market may alleviate Fe and Ca deficiencies and boost antioxidant status amongst Pakistani population [12], [13].

In beverage industry, quality assurance and shelf-life enhancement has become the biggest challenges. To prevent microbial spoilage or to retain optimal organoleptic properties of the drinks, synthetic preservatives are often incorporated as additives such as potassium metabisulphite or sodium bicarbonate, etc., though they are not only expensive but may also pose potential health risks if intake exceeds a certain time. In this context, scientific fraternity is now looking for some natural and cost-effectives means to preserve the beverages [14], [15]. Based on the need, plants with antimicrobial properties are now being tested by the researchers to replace the synthetic counterparts [16], [17].

In this regard, dried leaves of *Moringa oleifera,* native to Indian subcontinent and renowned as drumstick tree, contain varied micronutrients and phytochemicals that are employed to treat malnutrition and health related disparities [18], [19], [20]. Currently, a group of researchers explored a different perspective of moringa extract *i.e*. preservation of fresh fruit juices such as carrot, pineapple, and orange juices [21]. One of their peers, Prabakarana et al. [22] found that myricetin, quercetin, and hydroxybenzoic acids are the most important antimicrobial compounds existed in moringa leaf extract. Now, scientific fraternity is showing their keen interest in finding optimized level of moringa extract for varied food products [23]. In this regard, different extraction techniques are under the study to optimize the yield of antimicrobial compounds from plant matrix and numerous researchers endorsed ultrasonic method as the best proven technique than others [24].

Considering these facts, the aim of the study was to develop a naturally preserved fig drink, utilizing moringa leaf extract, to ensure the optimal provision of nutrients and sensory acceptability of the drink. Purposely, antimicrobial response of varied levels of moringa leaf extract need to be tested in fig drink, and compared with potassium metabisulphite that is taken as an optimal synthetic preservative in numerous studies as stated by Fazio et al. [25] and Pereira et al. [26].

## 2 Materials and Methods

### 2.1 Reagents and samples

Fig fruit (English Fig variety) was procured from Haramosh valley, Gilgit-Pakistan and *Moringa oleifera* (Techiman variety) was collected from the University of Agriculture Faisalabad-Pakistan. The moringa extract was prepared in the Laboratory of Food Processing and Preservation, Department of Food Science and Technology, GC-Women University Faisalabad Pakistan. For analysis purpose, all reagents and chemicals were purchased from Sigma Aldrich and Merck (Darmstadt, Germany). The key reagents include ethanol (CAS: 64-17-5), 2,2-diphenyl-1-picrylhy-drazyl (DPPH, CAS: 1898-66-4), 2,2′-azino-bis(3-ethylbenzothiazoline- 6-sulfonic acid) (ABTS, CAS: 30931-67-0), Trolox 6-hydroxy-2,5,7, 8-tetramethylchroman-2-carboxylic acid (C_14_H_18_O_4_, CAS 53,188-07-1), iron (II) sulfate heptahydrate (FeSO_4_.7 H_2_O, CAS Number: 7782-63-0), Folin-Ciocalteu phenol reagent, gallic acid ((HO)_3_C_6_H_2_CO_2_H, CAS: 149-91-7), sodium carbonate (Na_2_CO_3_, CAS 497-19-8), aluminium chloride (AlCl_3_, CAS-7446-70-0), Catechin (C_15_H_14_O_6_, CAS 154-23-4), sodium acetate (CH_3_COONa, CAS 127-09-3) and potassium chloride (KCl, CAS 7440-09-7).

### 2.2 Preparation of materials

#### 2.2.1 Development of fig fruit juice

To prepare fig fruit juice, ripe fresh fig fruits were peeled manually using a knife. Fig fruit and pulp portions were blended (Moulinex, France) following centrifugation at 3000 rpm for 10 min and the obtained supernatant was then filtered using muslin cloth for further use [7].

#### 2.2.2 Preparation of dried moringa leaf powder

The fresh *Moringa oleifera* leaves were harvested, separated from stalks, sorted to remove the yellow or brown leaves that may alter the colour of final juice. Then the sorted leaves were washed to eliminate dust, blanched at 45˚C for 30 sec and finally left to drain. Afterwards, the leaves were dried in air circulated oven at 50 °C for 5 h to prevent the loss of active ingredients. Lastly, the dried leaves were ground to fine powder using a hand milling machine [16].

#### 2.2.3 Extraction of moringa leaf extract (MLE)

The dried powder of moringa leaves were taken in beaker (500  mL) with 70 % ethanol with a solid to solvent ratio of 1:10. The mass transfer of polyphenols from moringa leaves to ethanol was carried out using ultrasonic probe (VCX-750, Sonics & Materials) at 20 kHz frequency under ambient conditions for 10 min [27]. The slurries obtained via ultrasonication process were filtered individually using Whatman No.1 filter paper. The filtrate attained from all samples was evaporated using rotary evaporator attached with a vacuum pump (Model VP18R, Lab tech) and a condenser system. Total solvent was dried at 60 °C on water bath under a vacuum pressure of 0.07 MPa. The obtained extract was then dried at room temperature in petri plates and then stored in an air-tight glass jars and kept at room temperature until added to the final juice [21], [27], [28].

#### 2.2.4 Processing technology of fig fruit-moringa composite juice

Firstly, fig fruit was blended with water in a ratio of 1:2, afterwards sugars and CMC was added along with moringa leaves extract at set concentrations or KMS as per the treatment plan followed by pasteurization at 80˚C for 10 min. Then, the prepared juice treatments were finally filtered using muslin cloth and cooled at 35˚C trailed by aseptic filling in sterilized glass bottles, leaving 2 cm headspace, and stored under refrigerator at 4 ± 1 °C. The prepared samples were proceeded further for different analyses over the storage up to one month at different intervals; 1st, 15th and 30th Day. (See Table 1.).

**Table 1:** Percent formulations of MLE based fig fruit drinks.

**Ingredients**	**Formulations**					
	**FM_0_K_0_**	**FM_0_K_0.2_**	**FM_5_K_0_**	**FM_10_K_0_**	**FM_15_K_0_**	**FM_20_K_0_**
**Fig fruit & water blend**	94.7	94.5	89.7	84.7	79.7	74.7
**Moringa Leave Extract**	−	−	5	10	15	20
**Potassium Metabisulfite (KMS)**	−	0.2	−	−	−	−
**Sugar**	5	5	5	5	5	5
**Carboxy** **Methylcellulose** **(CMC)**	0.3	0.3	0.3	0.3	0.3	0.3

† FM_0_K_0_: (Negative control) Fig Fruit Drink + Moringa Leaf Extract = 0 + KMS = 0; FM_0_K_0_._2_: (Positive control) Fig Fruit Drink + Moringa Extract = 0 + KMS = 0.2; FM_5_K_0_: Fig Fruit Drink + Moringa Leaf Extract = 5 % + KMS = 0; FM_10_K_0_: Fig Fruit Drink + Moringa Leaf Extract = 10 % + KMS = 0; FM_15_K_0_: Fig Fruit Drink + Moringa Leaf Extract = 15 % + KMS = 0; FM_20_K_0_: Fig Fruit Drink + Moringa Leaf Extract = 20 % + KMS = 0.

### 2.3 Free-radical scavenging assays

#### 2.3.1 DPPH scavenging ability

DPPH (1, 1-diphenyl-2-picrylhydrazyl) free radical capturing potential via active ingredients in the prepared beverage was assessed as per the protocol of Fatima et al. [34]. For this purpose, fresh DPPH solution was prepared by adding 0.004 g of DPPH radical in 100 mL of methanol. The resultant DPPH solution (1 mL) was added to test tube along with sample (25 µL) and vortexed. Afterwards, the solution was kept under dark for 15 min that allows active agents to scavenge DPPH radicals. Then, the absorbance of all the treatments were recorded at 517 nm using UV/VIS Spectrophotometer (CECIL CE 7200). The outcomes of the analysis were expressed as % DPPH by calculating through equation herein:

$$\mathrm{DPPHradical}\left(\%\right)=\frac{\mathrm{ControlOD}-SampleOD}{\mathrm{ControlOD}}\times 100$$

#### 2.3.2 ABTS [2, 2′-azino-bis (3-ethylbenzothiazoline-6-sulphonic acid) assay

The antioxidants in fig fruit-moringa drink samples scavenges ABTS free radicals by donating electrons, resultantly green colour of ABTS radical suppresses that is noted through a change in absorbance as elaborated by Ashfaq et al. [29]. Intentionally, 5 mL of ABTS solution was diluted with methanol until the attainment of absorbance i.e. 0.700 ± 0.005. Then the prepared treatment (20 µL) was mixed with ABTS solution (5 mL), and absorbance was recorded at 734 nm. The treatment value was then measured through standard curve of Trolox and represented as Trolox Equivalent (TE).

#### 2.3.3 FRAP (Ferric reducing antioxidant Power) assay

The antioxidant in the developed drink has the potential to transform colourless ferric ions into blue-coloured ferrous ions, resulted in increased optical density at 593 nm as studied by Ashfaq et al. [29]. To conduct this test, fresh FRAP reagent was prepared by mixing following solutions; 300 mM acetate buffer (25 mL), 10 mM TPTZ (2.5 mL) and 20 mM FeCl_3_·6H_2_O (2.5 mL). The reagents solution was then mixed with sample and 10 min rest was given under dark followed by absorbance determination at 593 nm. The obtained results were denoted as μM Fe (II)/g employing linear standard curve based on varied strengths of FeSO_4_.

### 2.4 Active ingredients quantification

#### 2.4.1 Total phenolics (TP)

The total phenolic acids in the developed fig fruit-moringa drink were assessed via Folin-Ciocalteu method [30]. Purposely, the treatment (30 µL) was mixed with Folin-Ciocalteu reagent (150 µL) and 7.5 % of Na_2_CO_3_ (120 µL) followed by mixing through vortex for 10 s and stay under darkness at 40 °C for 30 min. Afterwards, absorbance of the treatment was recorded at 765 nm via UV/VIS Spectrophotometer (CECIL CE 7200) and findings were stated as mg GAE/100 mL of drink.

#### 2.4.2 Total flavonoids (TF)

The flavonoids in the drink samples were determined using aluminium chloride colorimetric technique [31]. Accordingly, the treatment (25 µL) was mixed with 5 % aq. NaNO_2_ (10 µL) and stayed for 15 min at room temperature. Then, 1 M NaOH dissolved in distilled H_2_O (50 µL) along with 10 % aq. AlCl_3_ (15 µL) was also incorporated into this mixture. Finally, the absorbance was noted at 430 nm and data expressed as catechin equivalents (CE) after comparing with standard curve.

#### 2.4.3 Total anthocyanins (TA)

The total anthocyanins were determined by pH-differential spectrophotometry method as described by Ashfaq et al. [29]. Purposely, sample (50 μL) was separately mixed with sodium acetate (1 mL) and potassium chloride (1 mL), and pH values were adjusted to 1 and 4.5 by employing HCl, according. After that, centrifugation was carried out at 5000 rpm for 25 min at 4°C, and supernatants were collected in 96-well plate followed by absorbance measurement at 510 nm.

$$A=\left(A510\right)pH1-\left(A510\right)pH4.5$$

$$Anthocyanin\left(\frac{\mathrm{mg}}{\mathrm{mL}}\right)=\frac{A\times Molecularweight\times DF}{\varepsilon \times 1}$$

where, y = content of anthocyanins; A = absorbance; ε = extinction co-efficient; DF = dilution factor

### 2.5 Quality evaluation

The pH was analyzed using calibrated digital pH meter (Ino Lab 720, Germany) whose probe was inserted into the treatment and value was recorded once stable [32].

The total acidity of the prepared drinks was assessed as per the procedure described by Nawaz et al. [33]. For analysis, the drink (10 mL) was taken in a beaker along with distilled water (25 mL) and titrated with 0.1 N NaOH, carrying two drops of phenolphthalein indicator. The volume of NaOH consumed in neutralizing acidity of the drink was recorded in triplicates and represented as % citric acid equivalent.

$$\mathrm{Totalacidity}\left(\%\right)=\frac{\mathrm{AverageVol}.ofNaOH\times 0.1NNaOH\times Factormiliequivalentcitricacid(0.064)}{\mathrm{Weightofsample}}\times 100$$

The soluble solids in the drink samples were assessed via portable refractometer (TAMCO, Model No. 90021, Japan) as explicated by Fatima et al. [34]. Firstly, the refractive index of distilled water is checked to calibrate the refractometer then, the samples were placed on the lens of the refractometer and values were determined at 20 °C as Brix.

For the determination of reducing, non-reducing, and total sugars, Lane-Eynon Method was employed as per the protocol of Dos Santos et al. [35]. For reducing sugars, 5 mL of the sugar sample was diluted to 50 folds and pH was adjusted to 8, then this solution was transferred to a burette. For titration, 10 mL of Fehling’s A: Fehling’s B solution in 1:1 ratio was taken in a conical flask along with water (25 mL) and heated it till boiled, then 1 % methylene blue as an indicator was added that changed its colour to blue. On heating for 3 min, the mixture changed its colour to brick red (end point). In this way, titration was performed in triplicates and average value of sugar sample volume was taken. For non-reducing sugars, 5 mL of the sample was mixed with 3 mL of conc. HCl and stayed for 30 min at 68 °C, and pH was adjusted to 8 and diluted to 50 folds then the solution was transferred to a burette and titration done as mentioned earlier.

$$Reducing\;Sugars=\frac{\;100\times vb\times f\;}{M\;\times V}$$

$$Non\;Reducing\;Sugars=\frac{\;100\times vb\times 2\times f\;}{M\;\times V}\times 0.96$$

TotalSugars=ReducingSugars+NonReducingSugars

where, *Vb* = volume of the volumetric flask used (mL); *f* = Fehling’s solution factor; *M* = mass of sample in grams; *V* = diluted sample solution used in titration (mL) To quantify ascorbic acid, titration method was employed using 2,6-dichlorophenol indophenol dye, whose color is blue and alters to pink if ascorbic acid exists amongst the treatments [36]. Initially, the dye factor was calculated by standardizing dye solution against standard ascorbic acid in the presence of 3 % *meta*-phosphoric acid, and titrate volume noted as endpoint, indicating light pink color. Then, the sample was diluted with 3 % *meta*-phosphoric acid followed by filtration and titration against dye, till light pink. Here, volume of the dye used was noted and concentration was denoted as mg ascorbic equivalent to mL of dye solution.

### 2.6 Color

The color tonality of the drinks was analyzed using CIE-Lab colorimeter (CIELAB SPACE, Color Tech-PCM, USA) as per the guidelines of Yüceer and Caner [37]. The sample was placed in a plastic cup and light from the bottom of the cup captures the color of sample and values of L (lightness; 0 = an ideal black object, 100 = an ideal white object), a* (–a greenness; +a redness) and b* (–b blueness; +b yellowness) were displayed onto screen.

### 2.7 Microbial evaluation

The total aerobic bacteria (TAB) in the prepared samples were measured via the pour plate method [38]. All samples were serially diluted with sterile NaCl (0.85 %), then 1.0 mL of each dilution was plated in duplicate plates and plate count agar (Beijing Land Bridge Technology Co. Ltd., Beijing, China) was poured in these plates followed by solidification and inversion of plates then incubation was done at 37 °C for 48 ± 2 h, finally total aerobic bacteria was counted as CFU/mL. Further, viable yeasts and molds count were done through spread plate technique employing rose bengal agar (Beijing Land Bridge Technology Co. Ltd., Beijing, China) after incubation at 27 °C for 72 ± 2 h, followed by enumeration of total fungi as CFU/mL.

### 2.8 Sensory study

The prepared drinks were evaluated from 15 organoleptic assessors, aged between 23 to 40 years. The method used was nine-point hedonic scale ranged between extremely disliked; 1 and extremely liked; 9 [39]. The panelists were served with the drink samples in transparent cups (25 mL), that were labeled randomly and directed to rate for color, taste, odor, consistency, and after-taste. The drink was provided at room temperature under fluorescent light in separated booths to avoid biasness while rating. Additionally, the panelists were supplied with mineral water to rinse their palate during the tasting session. Further, consumer acceptability was calculated using the results of the above-mentioned aspects to deduce the consumer preferable drink.

### 2.9 Data analysis

The obtained results were compiled and subjected to statistical analysis Statistix 8.1 to determine the significance of each parameter [40]. Based on treatment and storage variables, two-way ANOVA under completely randomized design was implemented on the study. The variance amongst the treatment means was quantified through Tukey’s honest significant difference (HSD) test. All the analyses were noted in triplicates except sensory studies (n = 15).

## 3 Results and discussion

### 3.1 Antioxidative potential of MLE based fig fruit drinks

Inclusion of different levels of ultrasound assisted moringa extract and optimum level of potassium metabisulphite (KMS) in fig drink indicated considerable variations (p<0.05) with respect to free radical scavenging ability, as expressed by DPPH-, ABTS-, and FRAP tests (Table 2). It was observed that the FM_0_K_0_ (negative control) showed maximum existence of free radicals throughout storage. However, as level of moringa extract (natural preservative) was exceeded from 5 to 20 %, the control on free radicals started improving gradually, but still less than FM_0_K_0.2_ (positive control) especially against DPPH and ABTS radicals, however moringa extracts based treatments and FM_0_K_0.2_ results were comparable with respect to FRAP reducing power. This trend was in coherence with the presence of polyphenols, flavonoids, and anthocyanins in the prepared drinks that also indicated substantial differences (p<0.05) amongst all the formulations. Regarding storage, functional ingredients and their antioxidant potential in the prepared drinks expressed significant loss (p<0.05) under refrigeration. Nevertheless, the maximum decline in active ingredients was noted for FM_0_K_0_ followed by FM_5_K_0_. Further, as the concentration of moringa extract increases, the preservation potential also started rising accordingly. Besides, minimum deviation in drink storage quality was recorded for FM_0_K_0.2_, carrying KMS as synthetic preservative.

**Table 2:** Antioxidative potential of MLE based fig fruit drinks during storage.

	**Formulations**	**Storage intervals (Days)**			**Means**
		**1st**	**15th**	**30th**	
**DPPH (%)**	**FM_0_K_0_**	50 ± 1jk	39 ± 1kl	33 ± 0 l	39 ± 1e
	**FM_0_K_0.2_**	57 ± 3hi	54 ± 4i	50 ± 5ij	54 ± 4d
	**FM_5_K_0_**	73 ± 3cdef	67 ± 2 fg	61 ± 3gh	67 ± 3c
	**FM_10_K_0_**	81 ± 4abc	76 ± 6bcde	69 ± 4ef	75 ± 5b
	**FM_15_K_0_**	83 ± 3ab	77 ± 4bcd	71 ± 6def	77 ± 3ab
	**FM_20_K_0_**	85 ± 3a	80 ± 7abc	75 ± 6bcde	80 ± 6a
**Means**		71 ± 3a	65 ± 4b	60 ± 4c	
**ABTS (mg/mL)**	**FM_0_K_0_**	4 ± 0i	3.3 ± 0.2i	2.9 ± 0i	3.3 ± 0.1e
	**FM_0_K_0.2_**	6.6 ± 0.3abcd	6.4 ± 0.3bcd	6.1 ± 0.3cde	6.4 ± 0.3b
	**FM_5_K_0_**	5.2 ± 0.2 fg	4.8 ± 0.2gh	4.4 ± 0.2 h	4.8 ± 0.2d
	**FM_10_K_0_**	5.1 ± 0.2fgh	4.9 ± 0.3gh	4.6 ± 0.2gh	4.9 ± 0.2d
	**FM_15_K_0_**	6.2 ± 0.3cde	5.9 ± 0.2de	6.7 ± 0.3de	5.9 ± 0.3c
	**FM_20_K_0_**	7.3 ± 0.4a	7 ± 0.2ab	6.7 ± 0.3abc	7.0 ± 0.3a
**Means**		5.6 ± 0.2a	5.4 ± 0.2b	5.1 ± 0.2c	
**FRAP (mM/mL)**	**FM_0_K_0_**	0.26 ± 0.01jk	0.21 ± 0.01kl	0.16 ± 0.01 l	0.21 ± 0.01f
	**FM_0_K_0.2_**	0.37 ± 0.02gh	0.34 ± 0.02hi	0.29 ± 0.01ij	0.33 ± 0.02e
	**FM_5_K_0_**	0.46 ± 0.01de	0.39 ± 0.02fgh	0.34 ± 0.02hi	0.4 ± 0.02d
	**FM_10_K_0_**	0.53 ± 0.03bc	0.46 ± 0.03de	0.41 ± 0.02efg	0.46 ± 0.03c
	**FM_15_K_0_**	0.54 ± 0.03b	0.49 ± 0.02bcd	0.48 ± 0.02def	0.49 ± 0.02b
	**FM_20_K_0_**	0.6 ± 0.03a	0.54 ± 0.03b	0.48 ± 0.02 cd	0.54 ± 0.02a
**Means**		0.46 ± 0.02a	0.41 ± 0.02b	0.35 ± 0.02c	

*Values containing different alphabets are significant (*p* < 0.05), ** Two-way ANOVA followed by Tukey’s HSD multiple comparison tests, ***Means ± SD (n = 3).

† FM_0_K_0_: (Negative control) Fig Fruit Drink + Moringa Leaf Extract = 0 + KMS = 0; FM_0_K_0_._2_: (Positive control) Fig Fruit Drink + Moringa Extract = 0 + KMS = 0.2; FM_5_K_0_: Fig Fruit Drink + Moringa Leaf Extract = 5 % + KMS = 0; FM_10_K_0_: Fig Fruit Drink + Moringa Leaf Extract = 10 % + KMS = 0; FM_15_K_0_: Fig Fruit Drink + Moringa Leaf Extract = 15 % + KMS = 0; FM_20_K_0_: Fig Fruit Drink + Moringa Leaf Extract = 20 % + KMS = 0.

‡ DPPH = 1, 1-diphenyl-2-picrylhydrazyl, ABTS = 2, 2′-azino-bis (3-ethylbenzothiazoline-6-sulphonic acid), FRAP = Ferric Reducing Antioxidant Power.

**Fig. 1:**
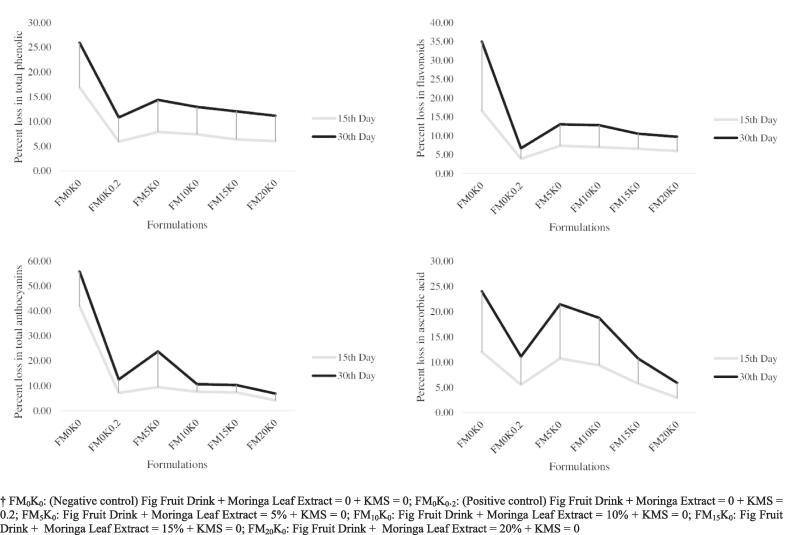
**Percent loss of functional ingredients in MLE based fig fruit drinks.** † FM_0_K_0_: (Negative control) Fig Fruit Drink + Moringa Leaf Extract = 0 + KMS = 0; FM_0_K_0_._2_: (Positive control) Fig Fruit Drink + Moringa Extract = 0 + KMS = 0.2; FM_5_K_0_: Fig Fruit Drink + Moringa Leaf Extract = 5 % + KMS = 0; FM_10_K_0_: Fig Fruit Drink + Moringa Leaf Extract = 10 % + KMS = 0; FM_15_K_0_: Fig Fruit Drink + Moringa Leaf Extract = 15 % + KMS = 0; FM_20_K_0_: Fig Fruit Drink + Moringa Leaf Extract = 20 % + KMS = 0.

At the termination of the study (30th day), the maximum decline in antioxidant ability (measured via DPPH-, ABTS- & FRAP assay) was ranged between 18 & 38 %, i.e. for FM_0_K_0_ whilst, the minimum decline in antioxidants was recorded for FM_0_K_0.2_ with values varying from 7 to 22 %. Inclusion of moringa extract amongst treatments; FM_5_K_0_, FM_10_K_0_, FM_15_K_0_, FM_20_K_0_ demonstrated loss in antioxidant potential to be varying from 14 to 26, 9–23, 9–19, and 8–20 %, respectively. Moreover, the maximum loss in polyphenols was viewed for FM_0_K_0_ (26 %) followed by 14 % for FM_5_K_0_, 13 % (FM_10_K_0_), 12 % (FM_15_K_0_) and 11 % (FM_20_K_0_ & FM_0_K_0.2_). In terms of flavonoids, the maximum loss was in FM_0_K_0_ i.e. 35 % followed by 13 % (FM_5_K_0_), 13 % (FM_10_K_0_), 11 % (FM_15_K_0_), and 10 % (FM_20_K_0_). However, the maximum stability in flavonoids was viewed in case of FM_0_K_0.2_ i.e. 7 %. The total anthocyanins were maximally reduced in FM_0_K_0_ (56 %) trailed by FM_5_K_0_ (24 %), FM_0_K_0.2_ (13 %), FM_10_K_0_, FM_15_K_0_ and FM_20_K_0_ (Table 2, Table 3 and Fig. 1).

**Table 3:** Functional components of MLE based fig fruit drinks during storage.

	**Formulations**	**Storage intervals (Days)**			**Means**
		**1st**	**15th**	**30th**	
**TP (mg GAE/mL)**	**FM_0_K_0_**	61 ± 3hi	41 ± 1ij	37 ± 2j	43 ± 2f
	**FM_0_K_0.2_**	50 ± 2 h	47 ± 3hi	45 ± 2hij	48 ± 2e
	**FM_5_K_0_**	71 ± 3ef	65 ± 3 fg	61 ± 3 g	65 ± 3d
	**FM_10_K_0_**	78 ± 4cde	72 ± 3def	68 ± 4 fg	73 ± 4c
	**FM_15_K_0_**	91 ± 2ab	85 ± 4bc	85 ± 4 cd	85 ± 4b
	**FM_20_K_0_**	95 ± 5a	90 ± 5ab	85 ± 4bc	90 ± 5a
**Means**		72 ± 3a	67 ± 3b	62 ± 3c	
**TF (mg CE/mL)**	**FM_0_K_0_**	14 ± 1f	11 ± 1 g	9 ± 1 h	11 ± 1e
	**FM_0_K_0.2_**	14 ± 1f	13 ± 0 fg	13 ± 1 fg	13 ± 1d
	**FM_5_K_0_**	22 ± 1abc	21 ± 1cde	20 ± 1e	21 ± 1c
	**FM_10_K_0_**	23 ± 1abc	21 ± 1bcde	20 ± 1de	21 ± 1bc
	**FM_15_K_0_**	23 ± 1ab	22 ± 1bcd	22 ± 1cde	22 ± 1ab
	**FM_20_K_0_**	24 ± 1a	23 ± 1abc	22 ± 1bcd	23 ± 1a
**Means**		20 ± 1a	19 ± 1b	17 ± 1c	
**TA (mg cy-3-rutinoside/mL)**	**FM_0_K_0_**	0.43 ± 0.02i	0.25 ± 0.01j	0.19 ± 0.01j	0.29 ± 0.01d
	**FM_0_K_0.2_**	0.56 ± 0.03 fg	0.52 ± 0.03gh	0.49 ± 0.02hi	0.52 ± 0.03c
	**FM_5_K_0_**	0.59 ± 0.01ef	0.48 ± 0.03hi	0.45 ± 0.02i	0.51 ± 0.02c
	**FM_10_K_0_**	0.66 ± 0.03bcd	0.61 ± 0.04def	0.59 ± 0.03ef	0.62 ± 0.04b
	**FM_15_K_0_**	0.68 ± 0.04abc	0.63 ± 0.03cde	0.61 ± 0.03def	0.64 ± 0.03b
	**FM_20_K_0_**	0.73 ± 0.03a	0.7 ± 0.03ab	0.68 ± 0.03abc	0.7 ± 0.03a
**Means**		0.61 ± 0.02a	0.53 ± 0.03b	0.50 ± 0.02c	

*Values containing different alphabets are significant (*p* < 0.05), ** Two-way ANOVA followed by Tukey’s HSD multiple comparison tests, ***Means ± SD (n = 3).

† FM_0_K_0_: (Negative control) Fig Fruit Drink + Moringa Leaf Extract = 0 + KMS = 0; FM_0_K_0_._2_: (Positive control) Fig Fruit Drink + Moringa Extract = 0 + KMS = 0.2; FM_5_K_0_: Fig Fruit Drink + Moringa Leaf Extract = 5 % + KMS = 0; FM_10_K_0_: Fig Fruit Drink + Moringa Leaf Extract = 10 % + KMS = 0; FM_15_K_0_: Fig Fruit Drink + Moringa Leaf Extract = 15 % + KMS = 0; FM_20_K_0_: Fig Fruit Drink + Moringa Leaf Extract = 20 % + KMS = 0.

‡ TP = Total Phenolics, TF = Total Flavonoids, TA = Total Anthocyanins.

Currently, Khan et al. [17] studied the impact of moringa extract 7 % on polyphenols, flavonoids, antioxidant activity of sugarcane juice. It was revealed from their study that moringa extract based treatments portrayed minor deviation in the afore-said aspects as compared to the control *i.e*. in line with the present study. Earlier, Hashemi et al. [21] found slight decrement in polyphenols and antioxidant activity of fresh sweet orange juice enriched with 10 % of each; moringa extract, ginger juice and beetroot juice as compared to control and the similar trend is viewed in the present study. Further, Hashemi et al. [16] preserved guava-whey juice by utilizing moringa extract up to 1.5 to 2 % and endorsed the current study that loss in antioxidant activity is directly proportional to degradation of functional ingredients that may occur because of oxidation reactions or enzymatic activity. Further, they elaborated that free radical chain reactions accelerate as the storage time exceeds.

### 3.2 Quality attributes of MLE based fig fruit drinks

Data in Table 4 indicated that formulations portrayed substantial distinctions (p<0.05) regarding TSS, pH, acidity, total-, reducing- & non-reducing sugars and ascorbic acid values. Similarly, on storage, considerable deviations (p<0.05) were recorded with respect to the afore-mentioned parameters except non-reducing sugar (Table 5). Further, all the treatments were different from each other with respect to CIE Lab* values however CIE ab* values were varying significantly from Day 1 to Day 30, except CIE L*.

**Table 4:** Quality attributes of MLE based fig fruit drinks.

**Formulations**	**Aspects**						
	**Total soluble solids (Brix)**	**pH**	**Titratable acidity (%)**	**Total Sugar (%)**	**Reducing sugar (%)**	**Non-reducing sugar (%)**	**AA (mg/mL)**
**FM_0_K_0_**	13 ± 0c	3.2 ± 0.1b	0.56 ± 0.02a	12.2 ± 0.5abc	4.8 ± 0.2a	7.4 ± 0.3d	0.22 ± 0.01a
**FM_0_K_0.2_**	15 ± 1a	3.4 ± 0.1ab	0.53 ± 0.02a	11.8 ± 0.5bc	4.0 ± 0.2b	7.8 ± 0.4 cd	0.34 ± 0.01a
**FM_5_K_0_**	13 ± 0c	3.4 ± 0.1ab	0.48 ± 0.02b	11.6 ± 0.4c	3.5 ± 0.1c	8.0 ± 0.3c	0.25 ± 0.01b
**FM_10_K_0_**	13 ± 1c	3.4 ± 0.2ab	0.45 ± 0.02c	12.3 ± 0.6ab	3.1 ± 0.1d	9.2 ± 0.4b	0.29 ± 0.01c
**FM_15_K_0_**	14 ± 1b	3.5 ± 0.1a	0.42 ± 0.01 cd	12.4 ± 0.5ab	3.0 ± 0.1d	9.5 ± 0.4b	0.31 ± 0.01d
**FM_20_K_0_**	15 ± 1a	3.5 ± 0.2a	0.39 ± 0.02d	12.9 ± 0.6a	2.6 ± 0.1e	10.3 ± 0.5a	0.33 ± 0.02e

*Values containing different alphabets are significant (*p* < 0.05), ** Two-way ANOVA (treatments expressed in Table 4 and storage demonstrated in Table 5) followed by Tukey’s HSD multiple comparison tests, ***Mentioned values are means of treatment means, ****Means ± SD (n = 3).

† FM_0_K_0_: (Negative control) Fig Fruit Drink + Moringa Leaf Extract = 0 + KMS = 0; FM_0_K_0_._2_: (Positive control) Fig Fruit Drink + Moringa Extract = 0 + KMS = 0.2; FM_5_K_0_: Fig Fruit Drink + Moringa Leaf Extract = 5 % + KMS = 0; FM_10_K_0_: Fig Fruit Drink + Moringa Leaf Extract = 10 % + KMS = 0; FM_15_K_0_: Fig Fruit Drink + Moringa Leaf Extract = 15 % + KMS = 0; FM_20_K_0_: Fig Fruit Drink + Moringa Leaf Extract = 20 % + KMS = 0.

‡ AA = Ascorbic Acid.

**Table 5:** Impact of storage on quality attributes of MLE based fig fruit drinks.

**Aspects**	**Storage intervals (Days)**		
	**1st**	**15th**	**30th**
**Total soluble solids (Brix)**	13 ± 1c	14 ± 1b	15 ± 1a
**pH**	3.5 ± 0.2a	3.4 ± 0.1a	3.3 ± 0.1b
**Titratable acidity (%)**	0.45 ± 0.02c	0.47 ± 0.02b	0.50 ± 0.02a
**Total Sugar (%)**	11.8 ± 0.5b	12.2 ± 0.5b	12.6 ± 0.6a
**Reducing sugar (%)**	3.0 ± 0.1c	3.4 ± 0.2b	4.1 ± 0.2a
**Non-reducing sugar (%)**	8.8 ± 0.4a	8.8 ± 0.4a	8.6 ± 0.4a
**AA (mg/mL)**	0.31 ± 0.01a	0.29 ± 0.01b	0.27 ± 0.01c

*Values containing different alphabets are significant (*p* < 0.05), ** Two-way ANOVA (treatments expressed in Table 4 and storage demonstrated in Table 5) followed by Tukey’s HSD multiple comparison tests, ***Mentioned values are means of storage means, ****Means ± SD (n = 3).

‡ AA = Ascorbic Acid.

Amongst the treatments, the maximum TSS was 15 ± 1 B i.e. of FM_0_K_0.2_ and FM_20_K_0_ followed by FM_15_K_0_ (14 ± 1 B) whereas minimum TSS was that of FM_0_K_0_ (13 ± 0 B), FM_5_K_0_ (13 ± 0 B) and FM_10_K_0_ (13 ± 1 B). In case of pH, the maximum value was noted for FM_20_K_0_ (3.5 ± 0.2) and FM_15_K_0_ (3.5 ± 0.1) trailed by FM_0_K_0.2_ (3.4 ± 0.1), FM_5_K_0_ (3.4 ± 0.1), FM_10_K_0_ (3.4 ± 0.2) and FM_0_K_0_ (3.2 ± 0.1). Alternatively, the maximum acidity was that of FM_20_K_0_ (0.39 ± 0.02 %) followed by FM_15_K_0_ (0.42 ± 0.01 %), FM_10_K_0_ (0.45 ± 0.02 %), FM_5_K_0_ (0.48 ± 0.02 %), FM_0_K_0.2_ (0.53 ± 0.02 %) and FM_0_K_0_ (0.56 ± 0.02 %). Total-, reducing- & non-reducing sugars in FM_0_K_0_ were (12.2 ± 0.5, 4.8 ± 0.2 & 7.4 ± 0.3 %) and (11.8 ± 0.5, 4.0 ± 0.2 & 7.8 ± 0.4 %) for FM_0_K_0.2_. Further, total- and non-reducing sugars in moringa extract based treatments demonstrated an inclining trend from 11.6 ± 0.4 and 8.0 ± 0.3 % to 12.9 ± 0.6 and 10.3 ± 0.5 % as the concentration of moringa extract increases from 5 to 20 %, respectively. On the other hand, moringa extract based treatments varying from 5 to 20 % indicated a gradual decreasing trend from 3.5 ± 0.1 to 2.6 ± 0.1 %, accordingly. Additionally, ascorbic acid (mg/mL) was maximally retained in FM_0_K_0.2_ (0.34 ± 0.01) followed by FM_20_K_0_ (0.33 ± 0.02), FM_15_K_0_ (0.31 ± 0.01), FM_10_K_0_ (0.29 ± 0.01), and FM_5_K_0_ (0.25 ± 0.01) whilst, the lowest was reported in FM_0_K_0_ (0.22 ± 0.01). At the termination of the study period, the percent loss of ascorbic acid was maximum in case of FM_0_K_0_ (24 %) whilst loss of ascorbic acid in FM_0_K_0.2_ and FM_15_K_0_ was at par, *i.e*. 11 %. In terms of moringa extract based treatments, the maximum loss was noted for FM_5_K_0_; 21 %, instead as the concentration increased up to 20 % (FM_20_K_0_), the losses reduced to minimum *i.e*. 6 %. During storage, the decline was observed in pH, non-reducing sugars, and ascorbic acids, accounting up to 6, 2 and 13 % whereas, TSS, acidity, total sugars, and reducing sugars showed an inclining trend by 13, 10, 6 and 27 % during 30 days of storage.

**Fig. 2:**
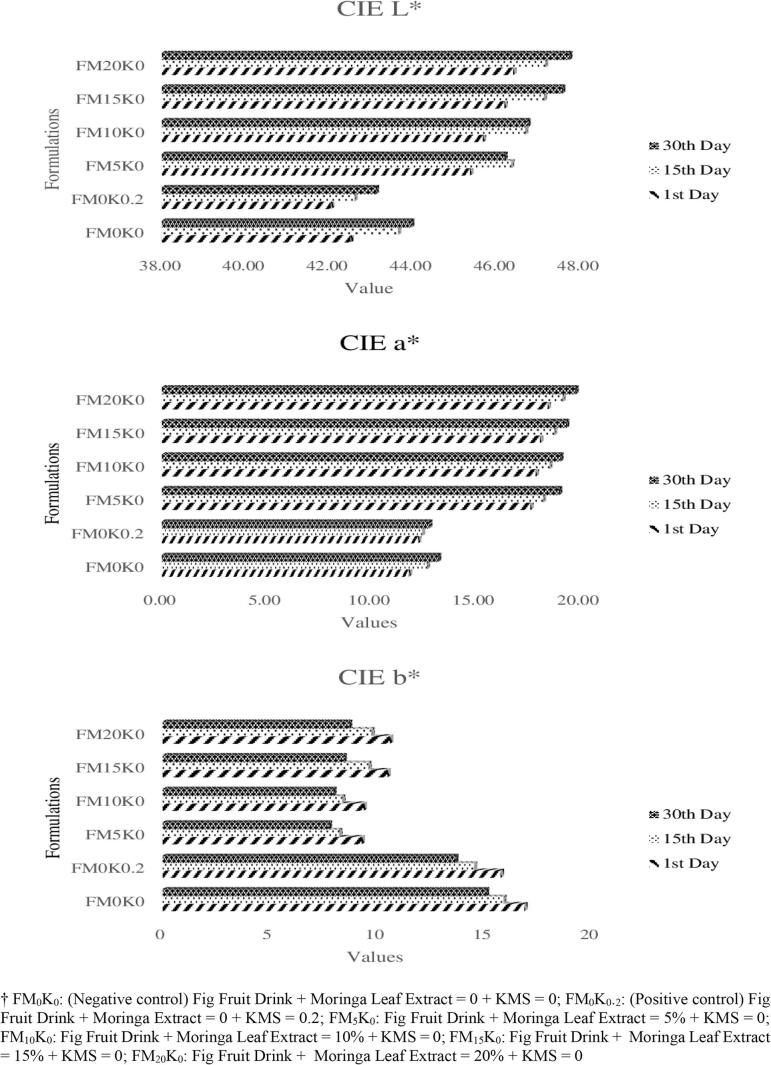
CIE Lab* values of MLE based fig fruit drinks. † FM_0_K_0_: (Negative control) Fig Fruit Drink + Moringa Leaf Extract = 0 + KMS = 0; FM_0_K_0_._2_: (Positive control) Fig Fruit Drink + Moringa Extract = 0 + KMS = 0.2; FM_5_K_0_: Fig Fruit Drink + Moringa Leaf Extract = 5 % + KMS = 0; FM_10_K_0_: Fig Fruit Drink + Moringa Leaf Extract = 10 % + KMS = 0; FM_15_K_0_: Fig Fruit Drink + Moringa Leaf Extract = 15 % + KMS = 0; FM_20_K_0_: Fig Fruit Drink + Moringa Leaf Extract = 20 % + KMS = 0.

Fig. 2 represents that CIE L* value increase with the rise in moringa extract concentrations from 46 ± 2 (FM_5_K_0_) to 47 ± 2 (FM_20_K_0_), with minimum values noted in FM_0_K_0_ and FM_0_K_0.2_ (43 ± 2). Furthermore, the CIE L* value increases from 45 ± 0 (Day 1) to 46 ± 1 (Day 30). In case of CIE a* value, the maximum value was that FM_0_K_0_ (13 ± 0) followed by FM_0_K_0.2_ (12 ± 1) whereas, as moringa extract concentration increases the CIE a* value increases from 18 ± 1 (FM_5_K_0_) to 19 ± 1 (FM_20_K_0_). However, on storage, the CIE a* value increases from 16 ± 0 (Day 1) to 17 ± 0 (Day 30). In terms of CIE b* value, maximum values were that of FM_0_K_0_ (16 ± 1) followed by FM_0_K_0.2_ (15 ± 1), moringa extract based treatments, rising from 8 ± 0 (FM_5_K_0_) to 10 ± 0 (FM_20_K_0_) whilst, storage presented significant decline from 12 ± 1 to 10 ± 0.

Increase in total sugar and reducing sugar might be due to hydrolysis of starchy materials or polysaccharides like pectin to simple sugars like glucose or fructose leading to increase in TSS as described by Thakur [15], who stated that ash gourd juice, preserved with different chemical preservatives, showed a significant rise (p ≤ 0.05) in reducing sugars from 0.15 to 0.48 % during storage. Additionally, Bal et al*.* [41] found increase in TSS in guava nectar from 13.17 to 13.48 B during storage.

The conversion of sugars to invert sugars alternatively decreases non-reducing sugars of the drink over the storage as elaborated by Majumdar et al. [42], who revealed decrease in non-reducing sugars in bottle gourd-basil juice from 8.27-7.59 % during storage. Further, solubilization of fig drink constituents or degradation of starches or sugars to organic acids on storage may result in accelerated acidity or decrease in pH as stated by Zakaria et al. [43], and Aderinola and Abare [44], who demonstrated that titratable acidity increased from 0.02 to 0.06 % in carrot-cucumber juice during storage. In the present study, the moringa extract based treatments has lesser acidity *i.e*. associated with acid binding ability of moringa extract as mentioned by Adeogun et al. [45] and Hashemi et al. [21].

The reduction of ascorbic acid in fig drink might be associated with its oxidation to dehydroascorbic acid through interaction with oxygen, and such losses are normally linked with processing procedures, storage duration, or exposure to light as stated by Fatima et al. [34], who reported reduction in ascorbic acid content in the functional drink to be ranged between 33 to 52 % during 30 days of refrigeration storage.

With respect to CIE Lab* value, the anthocyanin-based juices that have pink color, their Lab* values were varying between 27.44 and 45.53, −0.23 to 13.32, and 5.33 to 20.67, respectively. However, juices that have red color, their CIELab* values were in the range of 24.05 to 37.00 (L*), 8.49 to 17.05 (a*), and 2.87 to 11.94 (b*) *i.e*. in coherence with the current study findings [46].

### 3.3 Microbiological studies of MLE based fig fruit drinks

Moringa extract based fig drink formulations were assessed for total aerobic bacteria (TAB) and yeast & mold count (Y&MC) during refrigerated storage as shown in Table 6. Analysis of variance pertaining to TAB, and Y&MC of fig drinks revealed momentous impact (p<0.05) on treatments, storage, and their interactions. Amongst treatments, the maximum TAB was reported in FM_0_K_0_; 2.7 × 0.2 log CFU/mL however, the minimum TAB was reported in FM_0_K_0.2_ as 43 ± 2 log CFU/mL. Further, as the concentration of moringa extract increases, the control on TAB increases, varied between 2.5 × 0.1 log CFU/mL (FM_5_K_0_) to 2.2 × 0.1 log CFU/mL (FM_20_K_0_). Regarding storage, the TAB values progress from 1.5 × 0.1 log CFU/mL (Day 1) to 3.1 × 0.2 log CFU/mL (Day 30), the similar trend is expressed by Fatima et al. [34].

**Table 6:** Microbial evaluation of MLE based fig fruit drinks.

	**Formulations**	**Storage intervals (Days)**			**Means**
		**1st**	**15th**	**30th**	
**TAB (log CFU/mL)**	**FM_0_K_0_**	1.6 × 0.1gh	2.9 × 0.2c	3.5 × 0.2a	2.7 × 0.2a
	**FM_0_K_0.2_**	1.4 × 0.1i	2.1 × 0.1 fg	2.7 × 0.2 cd	2.1 × 0.1de
	**FM_5_K_0_**	1.6 × 0.1hi	2.7 × 0.1e	3.2 × 0.2ab	2.5 × 0.1b
	**FM_10_K_0_**	1.6 × 0.1hi	2.6 × 0.1e	3.1 × 0.2bc	2.4 × 0.1bc
	**FM_15_K_0_**	1.5 × 0.1hi	2.5 × 0.1ef	3.0 × 0.2c	2.3 × 0.1 cd
	**FM_20_K_0_**	1.5 × 0.1i	2.2 × 0.1 g	2.9 × 0.2d	2.2 × 0.1e
**Means**		1.5 × 0.1c	2.5 × 0.1b	3.1 × 0.2a	
**Y&M (log CFU/mL)**	**FM_0_K_0_**	1.3 × 0.1d	1.9 × 0.1b	2.5 × 0.1a	1.9 × 0.1a
	**FM_0_K_0.2_**	0.5 × 0.1 h	0.7 × 0.1gh	0.9 × 0.1 fg	0.7 × 0.1f
	**FM_5_K_0_**	1 × 0.1ef	1.4 × 0.1 cd	1.6 × 0.1c	1.3 × 0.1b
	**FM_10_K_0_**	0.8 × 0.1 fg	1.3 × 0.1d	1.4 × 0.1 cd	1.2 × 0.1c
	**FM_15_K_0_**	0.7 × 0.1gh	1 × 0.1ef	1.2 × 0.1de	1.0 × 0.1d
	**FM_20_K_0_**	0.7 × 0.1gh	0.8 × 0.1 fg	1 × 0.1ef	0.8 × 0.1e
**Means**		0.8 × 0.1c	1.2 × 0.1b	1.4 × 0.1a	

*Values containing different alphabets are significant (*p* < 0.05), ** Two-way ANOVA followed by Tukey’s HSD multiple comparison tests, ***Means ± SD (n = 3).

† FM_0_K_0_: (Negative control) Fig Fruit Drink + Moringa Leaf Extract = 0 + KMS = 0; FM_0_K_0_._2_: (Positive control) Fig Fruit Drink + Moringa Extract = 0 + KMS = 0.2; FM_5_K_0_: Fig Fruit Drink + Moringa Leaf Extract = 5 % + KMS = 0; FM_10_K_0_: Fig Fruit Drink + Moringa Leaf Extract = 10 % + KMS = 0; FM_15_K_0_: Fig Fruit Drink + Moringa Leaf Extract = 15 % + KMS = 0; FM_20_K_0_: Fig Fruit Drink + Moringa Leaf Extract = 20 % + KMS = 0.

‡ TAB = Total Aerobic Bacteria; Y&M = Yeast and Mold.

Regarding treatments, the Y&MC expressed highest growth in FM_0_K_0_; 1.9 × 0.1 log CFU/mL whilst, the minimum expansion was found in FM_0_K_0.2_ (0.7 × 0.1 log CFU/mL). Further, as the concentration of moringa extract increases, the control on Y&M activity became stronger, decreasing from 1.3 × 0.1 to 0.8 × 0.1 log CFU/mL. The trend is in harmony with Oniha et al. [47], who found anti-fungal characteristics of moringa extract in *Carica papaya* samples. During storage, the Y&MC indicated an inclining trend from 0.8 × 0.1 to 1.4 × 0.1 log CFU/mL.

It was noticed that expansion of microbes was fast in control negative treatment; FM_0_K_0_ however, as the moringa extracts dose increases, the inhibition of microbes increases though, the maximum control was indicated in positive control treatment; FM_0_K_0.2_. It is thus noted that the presence of moringa extracts and KMS creates unfavourable conditions for the growth of microbes predominantly aerobic mesophiles, yeast, and mold. Further, earlier researchers also found that moringa extract ranged between 10 and 20 % has the potency to control microbial growth in beverages for 28 days storage. This inhibitory action is attributed to polyphenolic components in moringa extract, that hinders the growth of microbes by interfering in nutrients transportation that are inevitable for their functioning [17].

### 3.4 Sensory response of MLE based fig fruit drinks

**Fig. 3:**
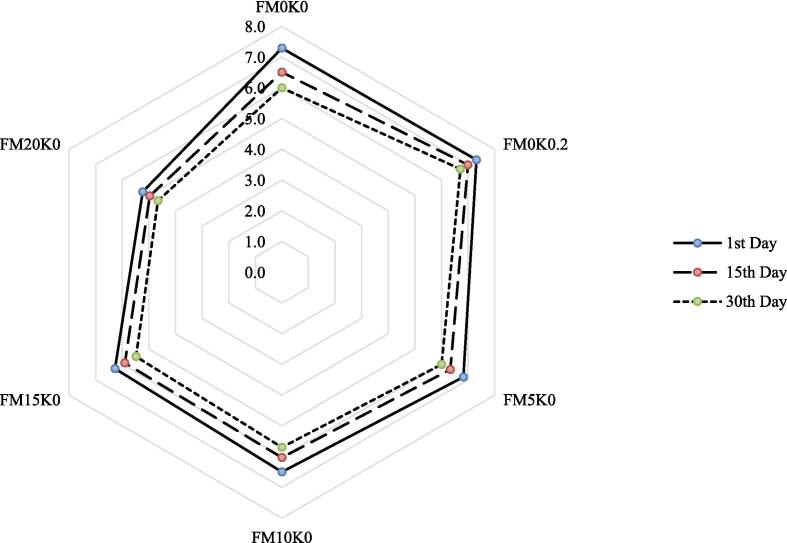
Consumer acceptability of MLE based fig fruit drinks.

Analysis of variance concerning to color, taste, odor, consistency, and after-taste of fig drinks depicted significant effects (p<0.05) on formulations and storage as shown in Table 7. The maximum scores for the sensory aspects were attained by FM_0_K_0.2_, that retained the sensory profile throughout storage followed by FM_0_K_0_, that indicated better scores at Day 1 than deviates to its maximum at Day 30. On the other hand, as the inclusion of moringa extract increases from 5 to 20 %, the sensory rating demonstrated a declining trend however, the maintenance of sensory perspectives was minimum over the storage. Similarly, low scores were reported over the storage for all the sensory responses. FM_0_K_0.2_ & FM_0_K_0_ rating for color were 7.2 ± 0.2 & 6.8 ± 0.2, taste (6.9 ± 0.1 & 6.5 ± 0.2), odor (7.1 ± 0.2 & 6.9 ± 0.2), consistency (7.0 ± 0.2 & 6.5 ± 0.1), and after-taste (6.9 ± 0.3 & 6.4 ± 0.2). The presence of moringa extract from 5 to 20 % resulted in decrease of color scores from 6.2 ± 0.3 to 5.3 ± 0.3, taste rating from 6.6 ± 0.3 to 5.3 ± 0.3, odor from 6.6 ± 0.3 to 5.0 ± 0.3, consistency from 6.5 ± 0.3 to 4.8 ± 0.2 and after-taste from 6.0 ± 0.2 to 4.3 ± 0.2. Regarding storage, the color scores decreased from 6.6 ± 0.2 to 5.8 ± 0.1, taste from 6.8 ± 0.2 to 5.9 ± 0.2, odor from 6.8 ± 0.1 to 5.9 ± 0.2, consistency from 6.5 ± 0.2 to 5.7 ± 0.1, and after-taste from 6.2 ± 0.2 to 5.4 ± 0.2. Conclusively, the FM_0_K_0.2_ indicated best acceptability (7.0 ± 0.3) followed by FM_0_K_0_ (6.6 ± 0.3). however, as the moringa extract concentration increases from 5 to 10, 15 and 20 %, the overall acceptability decreased from 6.4 ± 0.3 to 6.1 ± 0.3, 5.9 ± 0.3 and 5.0 ± 0.2. Throughout storage, the sensory response decreased up to 12.4 % from Day 1 to 30. (Fig. 3.).

**Table 7:** Sensory response of MLE based fig fruit drinks during storage.

	**Formulations**	**Storage intervals (Days)**			**Means**
		**1st**	**15th**	**30th**	
**Color**	**FM_0_K_0_**	7.5 ± 0.2a	6.7 ± 0.2bc	6.1 ± 0.2cdef	6.8 ± 0.2b
	**FM_0_K_0.2_**	7.5 ± 0.2a	7.1 ± 0.2ab	6.9 ± 0.2ab	7.2 ± 0.2a
	**FM_5_K_0_**	6.6 ± 0.3bcd	6.2 ± 0.1cdef	5.9 ± 0.1efgh	6.2 ± 0.2c
	**FM_10_K_0_**	6.3 ± 0.3cde	5.9 ± 0.3efgh	5.4 ± 0.1ghi	5.8 ± 0.2d
	**FM_15_K_0_**	6 ± 0.1defg	5.6 ± 0.3efghi	5.1 ± 0.1hi	5.7 ± 0.2d
	**FM_20_K_0_**	5.6 ± 0.1fghi	5.3 ± 0.3hi	5.1 ± 0.1i	5.3 ± 0.2e
**Means**		6.6 ± 0.2a	6.1 ± 0.2b	5.8 ± 0.1c	
**Taste**	**FM_0_K_0_**	7.3 ± 0.1a	6.4 ± 0.1cde	5.8 ± 0.4efg	6.5 ± 0.2b
	**FM_0_K_0.2_**	7.2 ± 0.1ab	6.8 ± 0.1abcd	6.5 ± 0.1bcd	6.9 ± 0.1a
	**FM_5_K_0_**	7 ± 0.2abc	6.6 ± 0.2abcd	6.3 ± 0.3de	6.6 ± 0.2ab
	**FM_10_K_0_**	6.9 ± 0.2abcd	6.5 ± 0.3abcd	6.1 ± 0.2def	6.5 ± 0.2b
	**FM_15_K_0_**	6.8 ± 0.4abcd	6.4 ± 0.3cde	5.1 ± 0.2efg	6.3 ± 0.3b
	**FM_20_K_0_**	5.6 ± 0.3fgh	5.3 ± 0.2gh	5.1 ± 0.2 h	5.3 ± 0.2c
**Means**		6.8 ± 0.2a	6.3 ± 0.2b	5.9 ± 0.2c	
**Odor**	**FM_0_K_0_**	7.5 ± 0.2a	6.8 ± 0.1bcd	6.4 ± 0.2defg	6.9 ± 0.2a
	**FM_0_K_0.2_**	7.4 ± 0.1a	7.1 ± 0.1abc	6.8 ± 0.2bcde	7.1 ± 0.2a
	**FM_5_K_0_**	7.1 ± 0.3ab	6.5 ± 0.2defgh	6.2 ± 0.3bcde	6.6 ± 0.3b
	**FM_10_K_0_**	6.8 ± 0.1bcde	6.2 ± 0.1efgh	5.9 ± 0.3gh	6.3 ± 0.2c
	**FM_15_K_0_**	6.5 ± 0.1cdef	6.2 ± 0.3fgh	4.6 ± 0.1hi	6.1 ± 0.2c
	**FM_20_K_0_**	5.3 ± 0ij	5.1 ± 0.1jk	4.6 ± 0.1 k	5 ± 0.1d
**Means**		6.8 ± 0.1a	6.3 ± 0.2b	5.9 ± 0.2c	
**Consistency**	**FM_0_K_0_**	7 ± 0.1ab	6.4 ± 0.1bcde	6 ± 0.1	6.5 ± 0.1b
	**FM_0_K_0.2_**	7.3 ± 0.1a	7 ± 0.4ab	6.7 ± 0.1bc	7 ± 0.2a
	**FM_5_K_0_**	6.9 ± 0.3ab	6.4 ± 0.1bcde	6.1 ± 0.1cdefg	6.5 ± 0.2b
	**FM_10_K_0_**	6.5 ± 0.1bcd	6 ± 0.3defg	5.7 ± 0.1 fg	6.1 ± 0.2c
	**FM_15_K_0_**	6.3 ± 0.3cdef	5.9 ± 0.2efg	4.5 ± 0.1gh	5.9 ± 0.2c
	**FM_20_K_0_**	5.1 ± 0.2hi	4.8 ± 0.1i	4.5 ± 0.1i	4.8 ± 0.1d
**Means**		6.5 ± 0.2a	6.1 ± 0.2b	5.7 ± 0.1c	
**After-taste**	**FM_0_K_0_**	7.1 ± 0.2a	6.3 ± 0.3cde	5.7 ± 0.2efg	6.4 ± 0.2b
	**FM_0_K_0.2_**	7.1 ± 0.3a	7 ± 0.2ab	6.7 ± 0.3abc	6.9 ± 0.3a
	**FM_5_K_0_**	6.4 ± 0.2bcd	6 ± 0.1def	5.6 ± 0.1efgh	6 ± 0.1c
	**FM_10_K_0_**	6.1 ± 0.3cdef	5.6 ± 0.1fgh	5.3 ± 0.3ghi	5.6 ± 0.2d
	**FM_15_K_0_**	5.8 ± 0.1defg	5.5 ± 0.1fgh	4 ± 0.1hi	5.4 ± 0.2d
	**FM_20_K_0_**	4.6 ± 0.2ij	4.4 ± 0.1jk	4 ± 0.1 k	4.3 ± 0.1e
**Means**		6.2 ± 0.2a	5.8 ± 0.1b	5.4 ± 0.2c	

*Values containing different alphabets are significant (*p* < 0.05), ** Two-way ANOVA followed by Tukey’s HSD multiple comparison tests, ***Means ± SD (n = 15).

† FM_0_K_0_: (Negative control) Fig Fruit Drink + Moringa Leaf Extract = 0 + KMS = 0; FM_0_K_0_._2_: (Positive control) Fig Fruit Drink + Moringa Extract = 0 + KMS = 0.2; FM_5_K_0_: Fig Fruit Drink + Moringa Leaf Extract = 5 % + KMS = 0; FM_10_K_0_: Fig Fruit Drink + Moringa Leaf Extract = 10 % + KMS = 0; FM_15_K_0_: Fig Fruit Drink + Moringa Leaf Extract = 15 % + KMS = 0; FM_20_K_0_: Fig Fruit Drink + Moringa Leaf Extract = 20 % + KMS = 0.

During storage, decrease in color scores of the fig drink is associated with enhanced millard reaction, accelerating browning effect in the final product. Apart from this, polyphenols in fig drink reacts with enzymes, causing discoloration. Moreover, the inclusion of moringa extract alters the color of fig drink, resulting in brown tint. The better taste score of control fig drink might be due to the higher content of fig juice or absence of moringa extract *i.e*. astringent or acidic in taste. As the moringa extract concentration increases, the off-taste or after-taste effect increases correspondingly, this is in harmony with the present study. However, on storage, taste deterioration is associated with hydrolytic, oxidative, and non-enzymatic reactions within the drink. Moreover, the reason behind significant decline in odor rating of fig drink with the advancement in storage, might be linked with enhancement in hydrolytic reactions, leading to higher concentration of acidity or unpleasant volatile odor that on further storage may cause slight fermentation or gas production [17], [34], [48]. Previously, Bal et al. [41] found that increase in soluble sugars or TSS causes increase in viscosity and decrease in flow index that might leads to decreased consistency of fig drink during storage *i.e*. in agreement with the current study.

## 4 Conclusions

This study provides invaluable outcomes for a beverage industry, working to bring fig drink into the market or trying to explore the effective level of moringa extract (natural preservative), as a safe substitute of its synthetic counterpart. It was revealed that as the concentration of moringa extract increased up to 20 % in fig fruit drink, the negative deviations during storage declined, but consumer preference started declining at 20 % moringa extract level. Considering this, 15 % moringa extract indicated as the customers preferrable dose where bacteria and fungi are also reasonably controlled. Consequently, it could be inferred that inclusion of moringa extract @ 15 % offers a harmonising approach to improve the storage stability of fig fruit drink. For future vistas, the major antimicrobial compounds in fig-moringa drink need to be assessed via advanced technologies to comprehend their in-depth mechanism.

## CRediT authorship contribution statement

**Faiza Javed:** Writing – review & editing, Writing – original draft, Software, Formal analysis, Data curation, Conceptualization. **Saima Tehseen:** Writing – review & editing, Writing – original draft, Resources, Project administration, Formal analysis, Data curation, Conceptualization. **Faiza Ashfaq:** Writing – review & editing, Writing – original draft, Software, Formal analysis, Data curation, Conceptualization. **Aysha Sameen:** Writing – review & editing, Supervision, Project administration, Investigation, Formal analysis, Data curation. **Waseem Khalid:** Writing – review & editing, Validation, Software, Resources, Formal analysis, Data curation, Conceptualization. **Rizwana Batool:** Writing – review & editing, Validation, Software, Methodology, Investigation, Formal analysis. **Ahmed Bilal:** Writing – review & editing, Writing – original draft, Validation, Supervision, Project administration, Methodology, Investigation, Formal analysis. **Muhammad Zubair Khalid:** Writing – review & editing, Methodology, Investigation, Formal analysis, Data curation, Conceptualization. **Tawfiq Alsulami:** Writing – review & editing, Validation, Methodology, Investigation, Funding acquisition, Data curation. **Robert Mugabi:** Writing – review & editing, Validation, Funding acquisition, Formal analysis, Data curation, Conceptualization. **Gulzar Ahmad Nayik:** Writing – review & editing, Writing – original draft, Validation, Resources, Formal analysis, Data curation, Conceptualization.

## Declaration of competing interest

The authors declare that they have no known competing financial interests or personal relationships that could have appeared to influence the work reported in this paper.
